# Supplementary material for: Tunable room-temperature ferromagnetism in Co-doped two-dimensional van der Waals ZnO
Source: Nat Commun. 2021 Jun 25;12:3952. doi: 10.1038/s41467-021-24247-w (PMC8233323; doi:10.1038/s41467-021-24247-w)
Supplement: Supplementary file 1 — Supplementary Information [file 41467_2021_24247_MOESM1_ESM.pdf]

Supplementary Information:

Tunable room-temperature ferromagnetism in Co-doped two-dimensional van der Waals ZnO

Authors: Rui Chen[1,2], Fuchuan Luo[1,8], Yuzi Liu[3], Yu Song[2,4], Yu Dong[5], Shan Wu[2,4], Jinhua Cao[1,2], Fuyi Yang[1,2], Alpha N'Diaye[6], Padraic Shafer[6], Yin Liu[1,2], Shuai Lou[1,2], Junwei Huang[5], Xiang Chen[2,4], Zixuan Fang[1,8], Qingjun Wang[1,2], Dafei Jin[3], Ran Cheng[7], Hongtao Yuan[5], Robert J. Birgeneau[1,2,4], Jie Yao[1,2*]

Affiliations:

[1] Department of Materials Science and Engineering, University of California, Berkeley, California 94720, United States.

[2] Materials Sciences Division, Lawrence Berkeley National Lab, Berkeley, California 94720, United States.

[3] Center for Nanoscale Materials, Nanoscience and Technology Division, Argonne National Laboratory, Lemont, Illinois 60439, United States.

[4] Department of Physics, University of California, Berkeley, California 94720, United States.

[5] National Laboratory of Solid-State Microstructures, College of Engineering and Applied Sciences, and Collaborative Innovation Center of Advanced Microstructures, Nanjing University, Nanjing 210093, P. R. China.

[6] Advanced Light Source, Lawrence Berkeley National Laboratory, Berkeley, California 94720, United States.

[7] Department of Electrical and Computer Engineering, University of California, Riverside, California 92521, United States.

[8] National Engineering Research Center of Electromagnetic Radiation Control Materials, University of Electronic Science and Technology of China, Chengdu 610054, P. R. China.

*Correspondence to: yaojie@berkeley.edu.

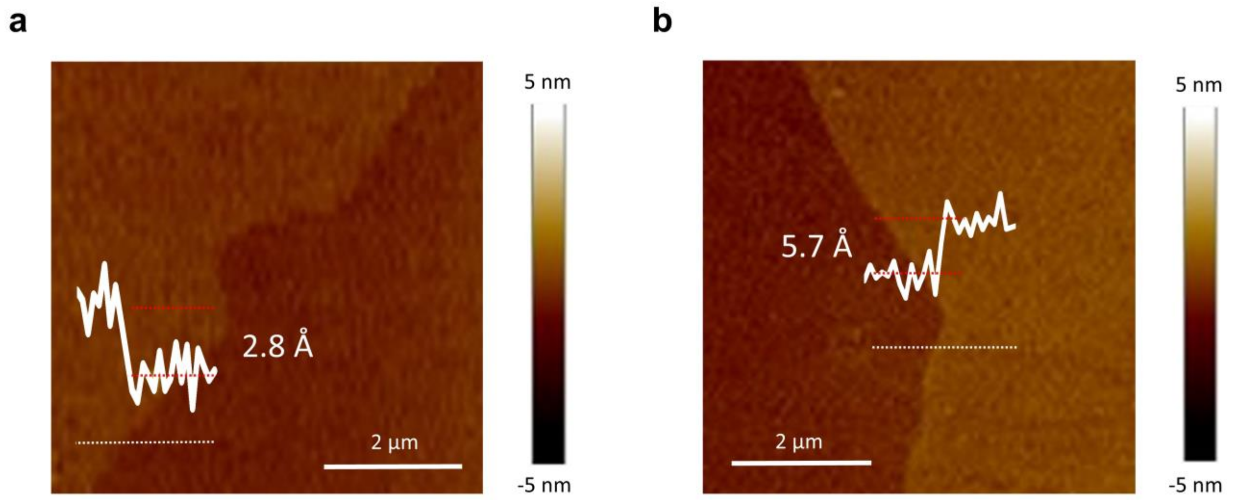

Supplementary Fig. S1 | AFM mapping of gZCO atomic layers. a, **b**, The morphology of monolayer (**a**) and bilayer (**b**) gZCO on SiO_2/Si substrates. Corresponding line scans (solid white lines) of the dashed white lines reveal the step height of the sample/substrate edge, being 2.8 Å (**a**) and 5.7 Å (**b**). Ultra-thin features of these samples are clearly resolved, along with atomically clean surfaces without contaminated 3D Co, cobalt oxides, and so on[32]. Note that these two samples are identified by MOKE, exhibiting ferromagnetic Kerr rotations at room temperature (shown in Fig. 1d and Fig. 3a).

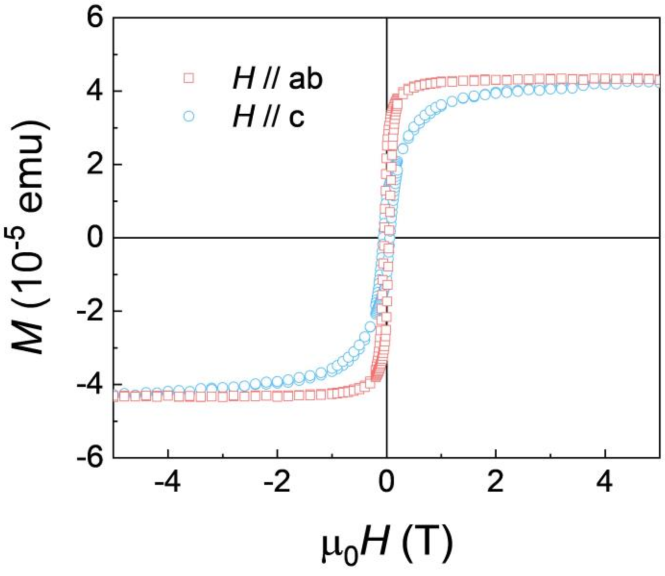

Supplementary Fig. S2 | *M-H* curves of 2D gZCO (11.9%)/rGO using SQUID. The *M-H* curves of 2D ZCO/rGO alternate layers are acquired by means of SQUID. External magnetic field is applied in and perpendicular to the film plane successively. A much smaller external field is required to fully saturate the magnetic moments when the field is along the ab plane (±1 T) instead of c-axis (±4.5 T). Therefore, we conclude that 2D ZCO displays an in-plane magnetic anisotropy.

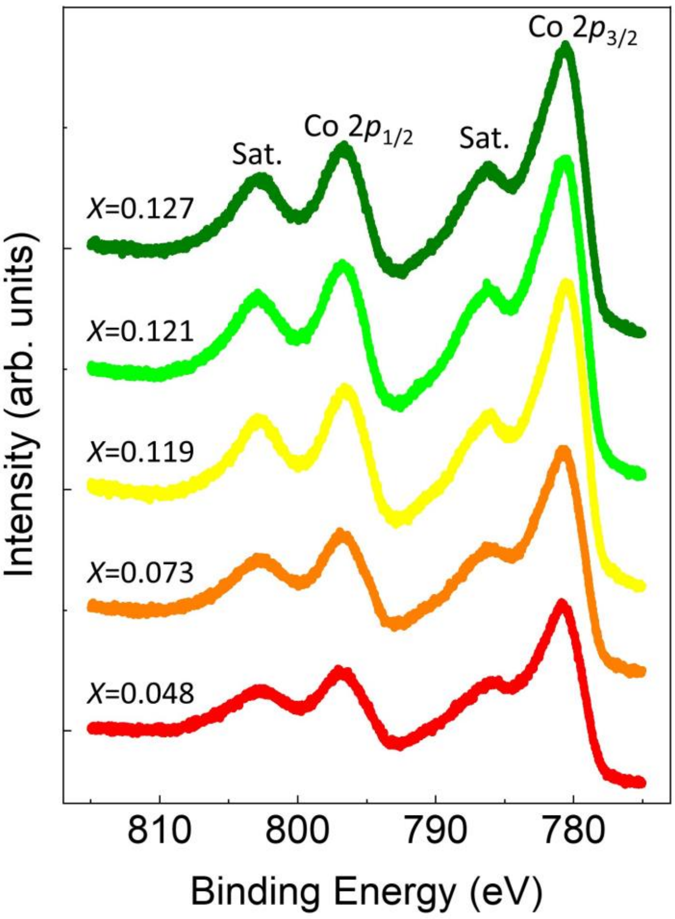

Supplementary Fig. S3 | XPS at Co *L* edges. XPS of the Co 2*p* levels in 2D gZCO/rGO alternate structures, displaying the fingerprint 2*p* binding energies (2$p_{3/2}$: 780.6 eV) and satellite (sat.) peaks of Co-O bonding, namely the +2 valence state of Co. This evidence coincides well with the XAS analyses (shown in Fig. 2a-c and Fig. 3b,c) that Co clusters can be ruled out in gZCO systems.

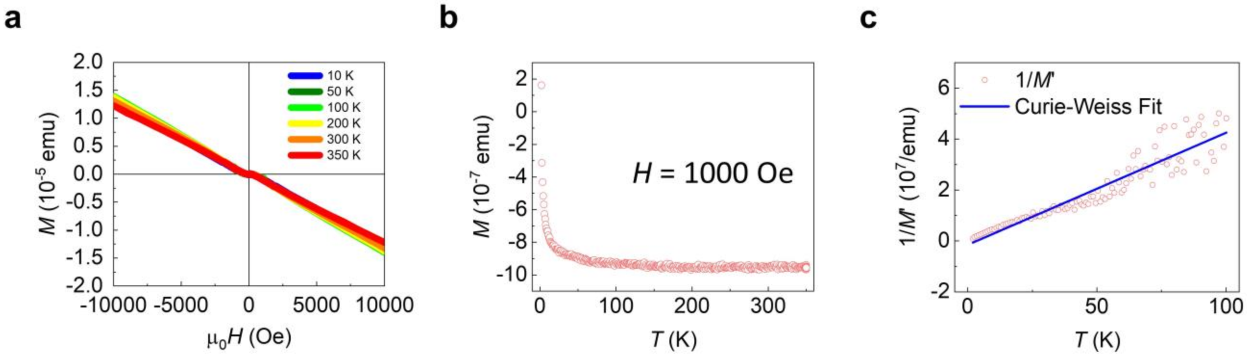

Supplementary Fig. S4 | SQUID measurements of gZnO (*x*=0) /rGO alternate structures. a, *M*-*H* curves with zero hysteretic loops showing typical paramagnetic and diamagnetic response in gZnO/rGO. **b**, *M*-*T* curves through FC. A magnetic field is applied in the sample plane with an amplitude of 1000 Oe. **c**, Fitting of the data in (**b**) at low temperatures according to Curie-Weiss law, exhibiting a representative paramagnetic feature. Note that *M′* is obtained after subtracting the negative background in (**b**). The negligible *M* and paramagnetic *M*-*T* feature rule out the ferromagnetism in both gZnO host and rGO template. We estimate that gZnO is paramagnetic and rGO is diamagnetic.

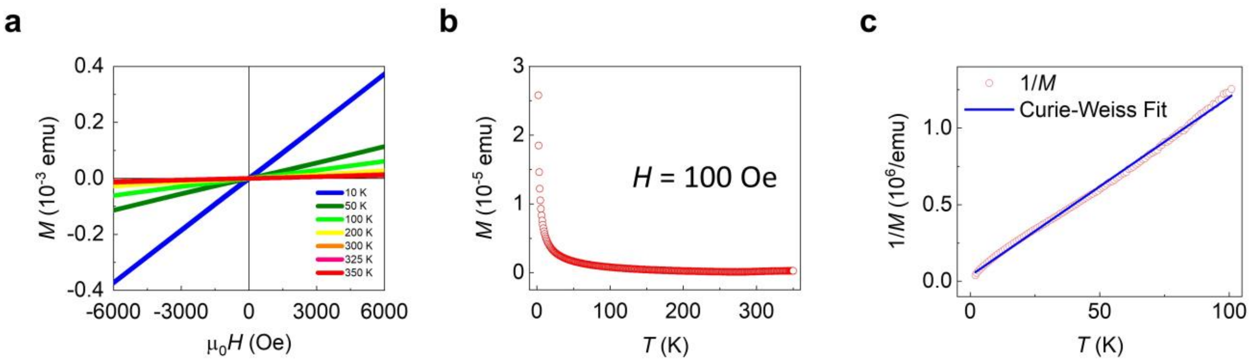

Supplementary Fig. S5 | SQUID measurements of the pure Co-precursor-synthesized control samples (*x*=1). a, Linear *M*-*H* curves with zero hysteretic loops showing fingerprint paramagnetic response. **b**, *M*-*T* curves through FC. A magnetic field is applied in the sample plane with an amplitude of 100 Oe. **c**, The Curie-Weiss law fitting of magnetic data in (**b**). It is evident from the linear fit in the low-temperature region (from 2 to 100 K) that our control samples (*x*=1) obey the Curie-Weiss law and exhibit no ferromagnetic couplings. The above control experiments explicitly rule out the cobalt oxides or Co nanoparticles as the possible ferromagnetic origin in 2D gZCO.

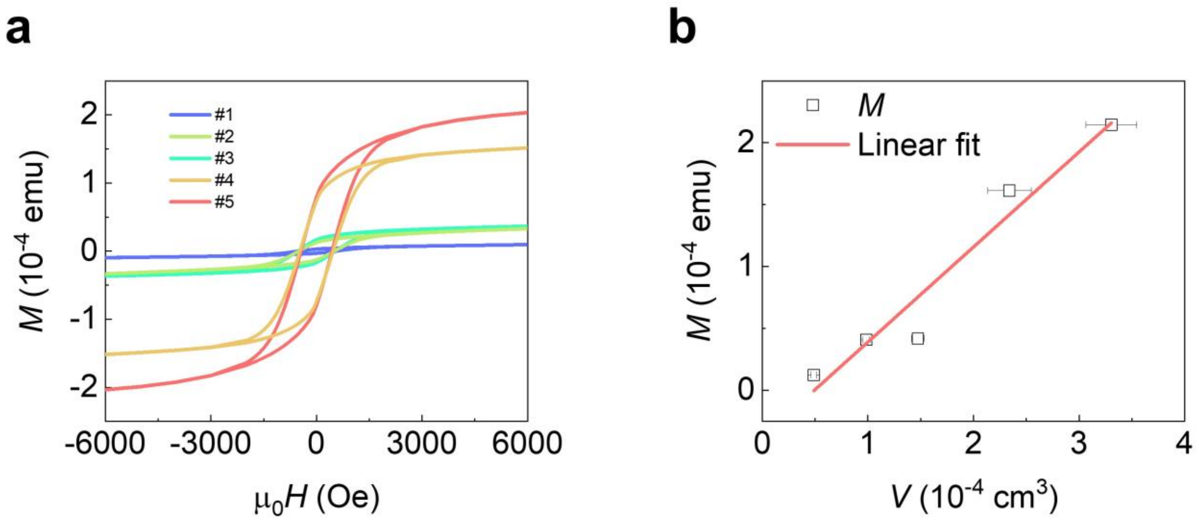

Supplementary Fig. S6 | Volume dependence of magnetic moments in 2D gZCO (*x*=0.119)/rGO. a, Ferromagnetic *M*-*H* loops at 300 K in five control samples with varying volumes. Consistent *M*-*H* behaviors (from sample #1 to #5) manifest reproducible room-temperature ferromagnetism in our synthesized 2D gZCO. **b**, A linear relationship between saturated magnetic moments M_s and sample volume *V*, which provides solid evidence that the SQUID-acquired magnetic signals do not originate from external impurities in substrates[14]. The red line is a linear fit to the data. Such a demonstration of linear M_s-*V* also sets a good example for the following investigations into 2D magnetic nanosheets using SQUID techniques.

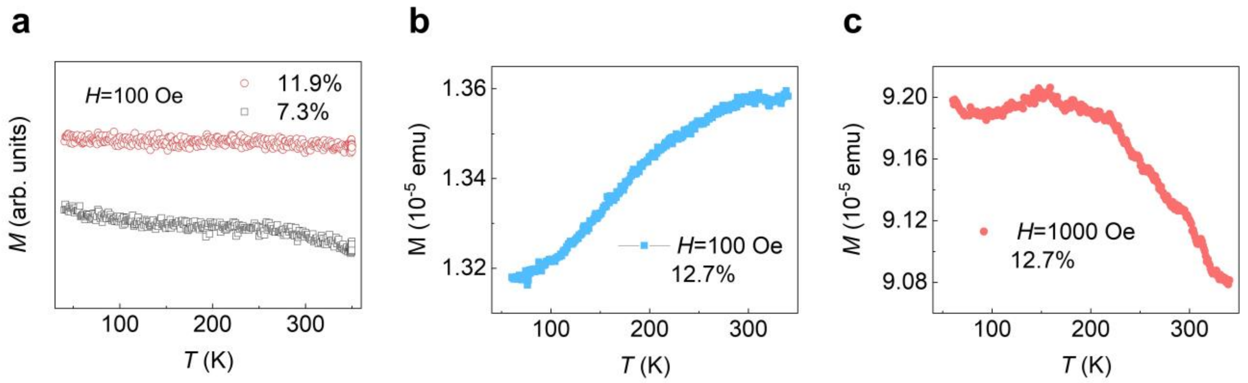

Supplementary Fig. S7 | *M*-*T* curves of 2D gZCO/rGO using SQUID. a, *M*-*T* curves for 7.3% (black square) and 11.9% (red sphere) Co doping cases. The external in-plane field is 100 Oe. For clarity, the two *M*-*T* curves are vertically offset. When Co doping is between 0 and 12.1%, a larger *M* is always evidenced at lower temperatures, due to the weaker thermal fluctuations and thus a stronger ferromagnetic ground state. This typical ferromagnetic *M*-*T* trend is in sharp contrast with the less ordered case (*x*=12.7%). **b**, *M*-*T* curves for 12.7% Co doping with a magnetic field of 100 Oe applied in the sample plane. An unambiguous suppression of magnetic ordering is observed at low temperatures because the occurrence of antiferromagnetic superexchange interaction competes with the ferromagnetic impurity-band-exchange interaction. Such evolution of *M*-*T* relations is consistent with that of *M*-*H* (shown in Fig. 4), as a function of Co doping level. **c**, *M*-*T* curves of the same sample in (**b**) with a higher field of 1000 Oe. In a larger external field, the less ordered spin couplings can be further driven into an ordered state, as evidenced by a stronger *M* at lower *T*.

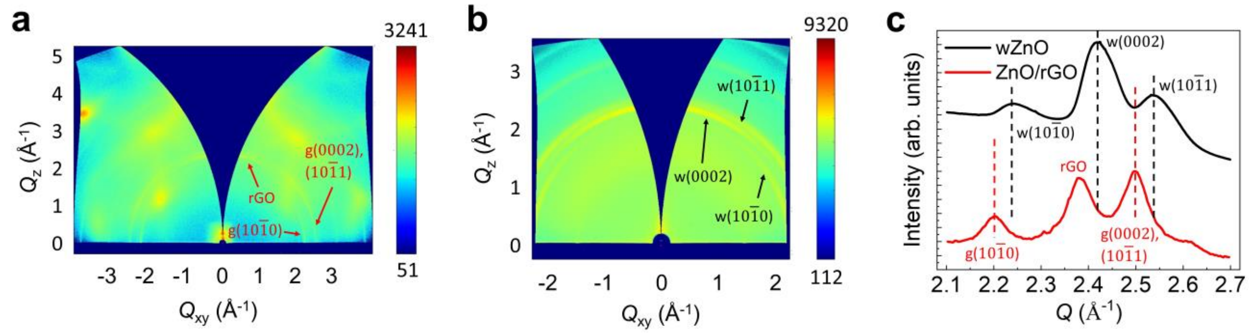

Supplementary Fig. S8 | GIWAXS measurements of ZnO/rGO and ZnO thin films. **a**, **b**, Scattering images from 2D ZnO nanosheets encapsulated by rGO (**a**) and ZnO thin films (**b**). Indices are provided for the most intense rings. The in-plane component of the momentum transfer vector Q is Q_{xy}; the out-of-plane component is Q_z. **c**, Integrated intensity values of the scattered rings in (**a**) and (**b**) as a function of Q, indicating characteristic graphitic and wurtzite $(10\bar{1}0)$, (0002) and $(10\bar{1}1)$ lattice planes in ZnO atomic layers and thin films, respectively. For example, the lattice spacing of $(10\bar{1}0)$ is 2.85 Å for ZnO/rGO while 2.81 Å for ZnO thin films. Therefore, our synthesized ZnO atomic layers, when intercalated into rGO, are unambiguously demonstrated to harbor a 2D graphitic lattice structure.

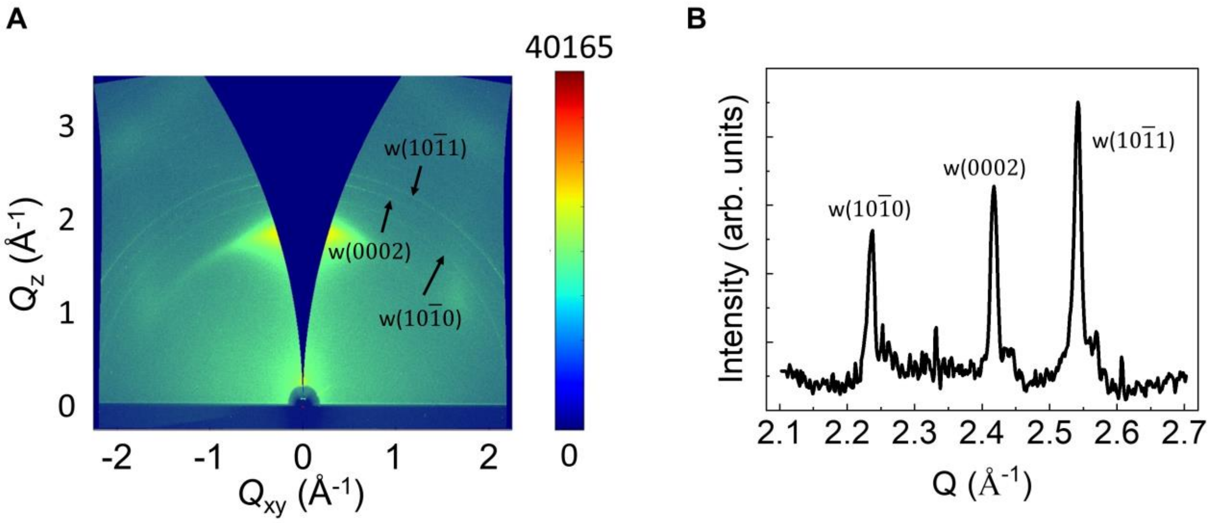

Supplementary Fig. S9 | GIWAXS measurements of Co: ZnO thin films. a, GIWAXS patterns of Co-doped ZnO thin films, displaying $(10\bar{1}0)$, (0002), and $(10\bar{1}1)$ lattice plane rings in hexagonal crystals. **b**, Integrated intensities for the scattering rings in (**a**), suggesting a wurtzite phase of ZnO host (lattice parameter of $(10\bar{1}0)$ plane: 2.81 Å), in stark contrast with the graphitic crystal structure of Co: ZnO atomic layers (shown in Fig. 2d).
